# Variations in maladaptive personality patterns and depressive symptoms’ severity across personality functioning profiles in a community sample

**DOI:** 10.1038/s41598-026-50285-9

**Published:** 2026-04-24

**Authors:** Iwona Nowakowska, Anna Zajenkowska, Katarzyna Czajkowska-Łukasiewicz, Kacper Łukasiewicz, Łukasz Gawęda, Jan Cieciuch

**Affiliations:** 1 Institute of Psychology, Maria Grzegorzewska University, Szczęśliwicka 40, 02-353 Warsaw, Poland; 2 Research Institute of Psychology, Vizja University, Warsaw, Poland; 3 Institute of Psychology, Polish Academy of Sciences, Warsaw, Poland

**Keywords:** Mental health, Depressive symptoms, Personality disorder, Dimensional psychopathology, Personality functioning, Diseases, Health care, Psychology, Psychology

## Abstract

Dimensional models of personality disorders, such as those proposed in the ICD-11 and the Alternative Model of Personality Disorders (AMPD) in the DSM-5, emphasize personality functioning and maladaptive personality traits, moving beyond the traditional categorical approach to personality disorders. Personality dysfunctions can co-occur with depressive symptoms; however, this relationship has been relatively understudied in the context of these dimensional frameworks.The purpose of the present study was to explore the heterogeneity of personality functioning profiles, including coexisting maladaptive traits and levels of depressive symptoms, in a community sample. The research sample was representative of the Polish adult population aged 18–65, comprising 1,030 participants (541 females, 489 males; *M*_*age*_ = 42.39, *SD*_*age*_ = 13.06). Data were collected via a survey administered through a research panel. We employed a two-step cluster analysis to examine patterns of psychopathological heterogeneity, followed by Kruskal–Wallis tests to explore differences between clusters. Personality functioning impairments clustered into groups that differed in the dysfunction severity. Specifically, we identified three groups corresponding to low, average, and high levels of self- and interpersonal impairment. As the level of impairment increased, additional subclusters emerged, exhibiting more diverse patterns of maladaptive personality domains. Furthermore, higher levels of personality pathology were associated with significantly greater severity of depressive symptoms. We discuss potential explanations for these findings and their implications for both psychotherapeutic and pharmacological interventions in individuals presenting with personality disorders and depressive symptoms.

**Supplementary Information:**

The online version contains supplementary material available at 10.1038/s41598-026-50285-9.

## Introduction

Personality disorders (PDs) are characterized by difficulties in self-functioning and/or interpersonal functioning, as defined by ICD-11. These disorders are persistent and enduring, and are marked by inflexibility and limited adaptability. They manifest through maladaptive thoughts, emotional experiences, and behavioral patterns, impacting multiple aspects of life^1^. PDs are not only a clinically relevant construct, they represent a significant public health concern in the general population, with prevalence estimates reaching 7.8%^2^ or 12.2%^3^. Depressive symptoms often co-occur with PDs^4^, as reflected in the high use of antidepressants among individuals with borderline PD^5^.

The contemporary understanding of PDs emphasizes dimensional diagnosis, which contrasts with the traditional categorical approach that groups symptomatic patterns into distinct disorders^6^. Until the introduction of DSM-5^6^ and ICD-11^1^, categorical classification predominated in psychiatry^7^. Nowadays, both DSM-5 and ICD-11 incorporate a dimensional perspective (in the case of DSM-5, it appears as an Alternative Model for Personality Disorders, AMPD, alongside the categorical approach). This perspective conceptualizes PDs along a spectrum from low to high levels of personality pathology. It may be especially valuable in non-clinical settings, such as prevention or screening, as it conceptualizes personality functioning as a continuum rather than relying on strict diagnostic cutoffs — allowing for the detection of subclinical manifestations of pathological personality traits^7^.

For this reason, the present study aimed to empirically examine the heterogeneity of maladaptive personality domain patterns across different levels of personality functioning impairment, using a dimensional framework of personality pathology in a community sample. Furthermore, given the high comorbidity between personality difficulties and depressive symptoms, sought to investigate how these patterns relate to levels of depressive symptoms. In the literature review below, we discuss how dimensional diagnostic approaches enhance understanding of personality impairments (both clinical and subclinical), as well as how the study of personality contributes to understanding depressive symptomatology in the general population. We then further elaborate on the rationale of the present study.

### Personality disorders: dimensional diagnosis

The dimensional diagnostic system for personality pathology involves two steps. First, the severity of impairment is assessed by evaluating both self and interpersonal functioning. DSM-5–based instruments, such as the Levels of Personality Functioning Scale, use a five-point scale ranging from no impairment to extreme impairment^6^. In ICD-11, PDs are categorized as mild, moderate, or severe based on the presence of essential features, while pronounced personality characteristics that fall short of clinical severity may be classified as “personality difficulty”^1,8^.

The second stage involves identifying up to five prominent maladaptive personality trait domains that further characterize the individual’s difficulties^9^. Impairment in personality functioning is generally associated with higher levels of maladaptive personality traits^10^. Three traits are shared by DSM-5 and ICD-11: Negative Affectivity (a propensity to experience negative emotions), Detachment (a tendency toward social isolation, introversion, and anhedonia), and Disinhibition (impulsivity and sensation-seeking). The fourth trait differs in labeling but represents a similar construct: in DSM-5 it is Antagonism (patterns of grandiosity, dominance, and aggression), whereas in ICD-11 it is Dissociality (a tendency to disregard social norms and others’ perspectives, including ruthlessness)^11^. The fifth trait is distinct between classifications: DSM-5 identifies Psychoticism (a tendency toward detachment from reality and unusual cognitive patterns), while ICD-11 identifies Anankastia (a narrow focus on right and wrong, accompanied by rigid perfectionism)^1,6^. These traits can be juxtaposed to theoretical frameworks regarding normal personality – Negative Affectivity can be understood as pathological Neuroticism, Detachment – maladaptively low Extraversion, Dissociality – as pathologically low Agreeableness, Disinhibition – maladaptively low Conscientiousness, and Anankastia – pathologically high Conscientiousness^11^. Such evidence may help understand the maladaptive personality patterns’ contribution to mental health outcomes in the general population, taking into account existing research on the Big Five and psychopathology. A dimensional view of PDs recognizes that individuals may possess varying degrees of maladaptive traits or dysfunctional patterns of thinking, feeling, and behaving — traits that are also characteristic of mood disorders. This perspective allows for a more precise understanding of how personality pathology and depressive symptoms overlap and interact, rather than treating them as wholly distinct clinical entities. Therefore, the dimensional approach is particularly relevant for understanding depressive symptoms, especially given the well-documented comorbidity between PDs and mood disorders^12^. Building on this, the present study aimed to examine the heterogeneity of maladaptive personality trait patterns across different levels of personality functioning impairment in a community sample. We further investigated how these patterns relate to depressive symptoms and maladaptive personality traits, with particular attention to Negative Affectivity. Unlike much of the existing research, which has focused on clinical samples or variable-level associations, this study adopts a person-centered approach to capture individual-level heterogeneity. A community-based sample was deliberately chosen, as it enables early identification of subclinical personality pathology and informs prevention-oriented interventions before clinical concerns emerge^13^.

### Personality and depressive symptoms

Based on clinical and empirical data, several theoretical perspectives have been proposed regarding the relationship between personality and depressive disorders^14,15^. These include: (1) shared underlying causal factors; (2) existence along a continuum; (3) personality characteristics preceding and signaling vulnerability to depression; (4) personality traits increasing the likelihood of developing depressive disorders; (5) personality influencing the expression and course of depression (pathoplastic effects); (6) certain personality features fluctuating with depressive episodes and being state-dependent; and (7) some traits emerging as lasting consequences or “scars” following depressive episodes. The boundaries between these explanations are often subtle^15,16^, and multiple models or hybrid frameworks may better capture the complexity of the relationship.

Determining whether PDs are primary to depression or vice versa is challenging, given symptom overlap^15^. Some authors suggest that personality functioning, maladaptive personality traits, and clinical outcomes such as depression may be conceptually interrelated, reflecting shared underlying mechanisms rather than distinct constructs^17^. Nevertheless, there is evidence that the measure of personality functioning (LPFS-BF 2.0) shows incremental validity beyond maladaptive traits in predicting well-being^18^. Furthermore, the abovementioned constructs serve distinct clinical functions: functioning predicts the general level of care and prognosis, whereas traits and depressive symptoms (life problems) help establish specific therapeutic goals and guide the selection of intervention techniques^17^.

There is also the possibility of different disorder trajectories because PDs may involve stable traits (trait PD) as well as episodic states (state PD)^19^. The latter represents transient personality dysfunction associated with other mental health difficulties, which can affect treatment. Notably, an intervention study of patients with clinical depression found that, after eight weeks of fluoxetine treatment, both depressive symptoms and the number of criteria met for borderline, narcissistic, dependent, and paranoid PDs were reduced^20^.

Furthermore, normal personality traits that correspond to the maladaptive personality traits are evidenced to play a role in depressive symptomatology. Neuroticism is consistently associated with higher depressive symptoms (for meta-analysis, see^21^. Individuals high in neuroticism tend to experience greater negative emotionality, such as sadness, irritability, and anxiety, which exacerbates depressive symptoms and may hinder treatment outcomes^22^. In contrast, extraversion is generally protective against depression, likely through its association with positive affect and increased social engagement^23,24^. Conscientiousness shows a negative relationship with depression, as traits such as self-discipline and adaptive coping reduce vulnerability to depressive symptoms^24^. Higher agreeableness may promote mental well-being by facilitating positive interpersonal relationships^25^, but its protective effects appear to depend on contextual factors such as social support and interpersonal stressors^26^. Also, the findings based on the Alternative Model for Personality Disorders (AMPD) indicate that elevated levels of DSM-5 personality dysfunctions and stylistic traits are strongly related to depression^27^. Negative Affectivity is the most predictive of depressive symptomatology out of all maladaptive personality traits^28^, and depressivity is one of its facets^6^. Specifically, individuals with higher levels of this trait report greater sadness, anxiety, and hopelessness, indicating its role as a risk factor for depressive symptoms in non-clinical populations^29–32^. Also Detachment has been linked to depression through its association with social withdrawal and reduced interpersonal engagement^30–32^. Supporting this, Sellbom et al.^33^ found that lower Detachment was associated with greater psychological well-being, underscoring the protective role of social connectedness. Moreover, Watson et al.^34^ reported that disinhibited behavior predicts higher depression levels, particularly under conditions of social and personal instability. Consistently, DeYoung^35^ found that individuals high in Disinhibition report greater depressive symptoms and poorer emotion regulation, suggesting reduced emotional resilience. Dissociality is linked to increased depressive symptoms, potentially due to impaired interpersonal functioning, social conflict, and isolation^36,37^. Also high Anankastia predicts higher vulnerability to depression due to rigid self-standards and emotional dysregulation^38,39^. Hemmati et al.^40^ further suggested that maladaptive coping strategies linked to perfectionism increase the risk of depressive symptoms.

In reality, however, people rarely present with a single maladaptive trait; rather, personality is a complex interplay of multiple traits operating within a given level of functioning. Despite existing empirical findings, there remains a lack of comprehensive investigation into how overall personality dysfunction and associated trait profiles relate to the intensity of depressive symptoms in the general population.

### Current study

Although most individuals in the general population do not meet the clinical criteria for a PD diagnosis, subclinical levels of personality dysfunction and maladaptive traits are considerably more widespread^41^. Yet research examining the heterogeneity of such trait patterns across different levels of personality functioning impairment remains scarce. These patterns may vary substantially, contributing to both subclinical and clinical manifestations of psychopathology, as well as to associated difficulties such as depressive symptoms^19^. Existing research has primarily focused on clinical samples or on associations between variables rather than on the heterogeneity of individual-level patterns. A community-based sample provides a critical foundation for mental health prevention and promotion^13^. Based on our previous findings from this sample^41^, three distinct levels of personality functioning impairment can be identified in a community sample, each associated with increasing severity of psychopathology. In the present study, we examine whether further subclusters of maladaptive traits emerge within these previously established impairment levels.

Identifying subclusters of maladaptive personality traits across levels of personality functioning impairment captures meaningful heterogeneity that variable-centered methods often obscure. This approach mirrors the sequential logic of dimensional diagnosis, which begins with assessing the severity of personality impairment and proceeds to identifying maladaptive trait profiles. Conducting subcluster analysis within a single theoretical framework enhances interpretability and yields clinically meaningful results. To the best of our knowledge, this is the first study to examine subclusters of maladaptive traits across clusters of personality functioning impairment severity.

Furthermore, we hypothesized that subclusters exhibiting greater personality functioning impairment and higher levels of maladaptive traits would report more severe depressive symptoms (H1), consistent with prior findings^31^. Our analytic choice is appropriate to our research goals given that cluster analysis groups individuals based on shared trait configurations rather than isolated trait levels, allowing the detection of latent subpopulations with distinct psychological profiles. Person-centered approaches have been shown to be useful for identifying subgroups with different risk patterns and intervention needs^42^. Moreover, personality traits are multidimensional, and averaging across individuals can mask clinically relevant differences that contribute to psychopathology^25^. By modeling trait constellations, cluster analysis offers a more ecologically valid representation of personality profiles and can improve risk stratification^16^. This approach allows us to capture the heterogeneity of personality pathology patterns observed in practice but often overlooked in dimensional models, enhancing interpretability of the contribution of maladaptive traits to comorbid depressive symptoms.

## Method

### Procedure

The study was questionnaire-based, and conducted online in September and October 2022. Online recruitment enabled us to reach a large community sample. A research panel ReaktorOpinii.pl (part of Pollster Research Institute in Poland) was responsible for data collection, and recruited participants out of an existing pool of its users. This research panel involves volunteers from the general Polish population interested in participating in a variety of business- and marketing-related, as well as scientific studies. The quality of the standards it applies is confirmed by its valid accreditations by ESOMAR (esomar.org), a global network of insights professionals, and PKPJA (a Polish programme of controlling quality of pollsters’ work in the country). The panel enables to recruit samples based on demands from the clients, and is experienced in conducting scientific research on representative samples of Poles.

The research panel contacted their panelists through emails, inviting them to participate in the study. The study was designed so as to recruit a sample representative in terms of the percentage of gender, age, size of place of residence and education level in the Polish population based on the census data from 2021^43^. This was achieved by setting relevant quotas and recruiting respondents until the desired structure of the sample was achieved.

The study was anonymous, and all participants provided informed consent. They were informed that discontinuation of the survey is possible anytime without any consequences. Given the specific topic of the study (mental health), on the last survey screen contact information to free-of-charge mental health helplines was provided. After completing the study, all participants received remuneration in the form of points that they could later exchange into money through the research panel. We excluded participants who answered incorrectly to any of 6 attention check questions (e.g., *Please mark the answer which indicates a name of a season of the year* or *Please mark the word “Monday”*). The materials and procedure were approved by the Maria Grzegorzewska Research Ethics Committee before the data collection began, approval no. 123/2022. The same dataset was used in previous publications^41,44^. The analysis presented here is complementary to them. The current research question is original.

### Measures

*Level of personality functioning (LPF)* as defined in the diagnostic guidelines for PD in the DSM-5 Section III was measured with the Polish adaptation of the Level of Personality Functioning Scale–Brief Form 2.0 (LPFS–BF 2.0)^45^, Polish version^18^. In the Polish adaptation study on a non-clinical sample, LPFS–BF 2.0 exhibited a robust two-factor latent structure reflecting self- and interpersonal functioning. It replicated the results obtained for the original version of the scale^45^. The Polish version demonstrated meaningful associations with indices of personality disorder severity, maladaptive personality traits, well-being, and the personality dimensions of agency and communion. Moreover, the LPFS–BF 2.0 showed incremental validity beyond the pathological trait domains in predicting global well-being. Reliability indices suggested satisfactory internal consistency for the tool, both for the total score (α = 0.84) and for the subscales assessing self (α = 0.82) and interpersonal functioning (α = 0.72)^18^.

Consequently, the tool consists of 12 items forming 2 subscales measuring Self and Interpersonal Functioning. There is also a possibility of computing the total score of personality functioning impairment^18,45^. For the Polish sample, the cut-off scores for severity benchmarks are assessed based on the total LPFS 2.0 score and are as follows: > 32 – mild personality impairment, > 36 – moderate personality impairment, > 40 – severe personality impairment, and > 44 – extremely severe personality impairment^46^. The respondents mark the degree to which each of the statements is true for them on a 4-point Likert scale (1 = definitely or very often untrue; 4 = definitely or very often true). The higher the score, the higher the level of impairment in a specific domain of functioning. The level of each of the domains was computed as a mean of relevant items, according to the key, as was the general score (only for introductory correlation analysis). In the current study, the Cronbach’s α indices were 0.88 for the Self Functioning, 0.80 for the Interpersonal Functioning, and 0.90 for the general score.

*Maladaptive personality traits according to a dimensional model of PDs proposed in ICD-11* were measured with the Polish adaptation of the Personality Inventory for ICD-11; PiCD^47^, Polish adaptation^48^. In the Polish adaptation study on a non-clinical sample, PiCD exhibited four-factor structure, confirming the original structure of the tool^47^, consisting of three unipolar dimensions—Negative Affectivity, Detachment, and Dissociality—and one bipolar dimension contrasting Anankastia and Disinhibition. All PiCD traits demonstrated theoretically expected associations with both PID-5 pathological traits and BFI-2 normative traits, as evidenced by correlational and factor-analytic results.

Importantly, as the adaptation study authors indicate^48^ the presence of a bipolar factor does not preclude interpretation in terms of five distinct domains, as bipolarity is not incompatible with the existence of distinguishable domains constituting its respective poles. Although Anankastia and Disinhibition jointly form a single bipolar dimension, they exhibit distinct and theoretically coherent patterns of associations with the LPFS dimensions and with the remaining PiCD domains. In light of these findings, as well as additional theoretical and empirical considerations^49,50^, the use of indicators representing the five ICD-11 trait domains appears to be the most justified approach, including from a clinical perspective. This is the approach recommended by the adaptation authors, also in the key to the measure. Reliability indices in the Polish adaptation study ranged from 0.77 to 0.87 (Mα = 0.82), reflecting acceptable levels of internal consistency and demonstrating comparability with the reliability estimates reported for the original version^47^.

Consequently, the tool consists of 60 items assessing the five domains for the ICD-11: Negative Affectivity, Disinhibition, Detachment, Dissociality, and Anankastia. Each item is rated on a scale from 1 (strongly disagree) to 5 (strongly agree). The level of each of the pathological domains was computed as a mean of relevant items, according to the key. Cronbach’s alphas for the subscales in the current study were: 0.91 for Negative Affectivity, 0.88 for Disinhibition, 0.87 for Detachment, 0.81 for Dissociality, and 0.79 for Anankastia.

*Depressive symptoms* were measured with Polish adaptation of Patient Health Questionnaire (PHQ-9^51^; Polish adaptation^52^ psychometrically tested on a mixed clinical and non-clinical sample. Identically to the original version, the Polish adaptation has a unidimensional structure, and showed external validity (strong correlations with Beck Depression Inventory, *rho* = 0.92; *p* <.001, and with the Hamilton Depression Rating Scale, *rho* = 0.87; *p* <.001). The reliability in the adaptation study was α = 0.88. The tool consists of 9 items providing a 0 to 27 severity score. For the Polish adaptation, the cut-off point for maximizing sensitivity and specificity of depression diagnosis is > 12^52^. The respondents mark the degree to which each of the statements is true for them on a 4-point Likert scale (0 = not at all; 3 = nearly every day in the last 2 weeks). The general score was computed as the sum of items. In the current study, the Cronbach’s α was 0.89.

### Participants

We recruited *N* = 1030 participants, aged 18–65 (*M* = 42.39; *SD* = 13.06); 541 (52.5%) biologically women, 489 (47.5%) biologically men. Sample size was determined as a typical sample for a representative study in Poland. Three hundred eighty-three respondents (37.2%) were village inhabitants, 213 (20.7%) lived in a town with less than 49 999 inhabitants, 121 (11.7%) in a town with 50 000–99 999 inhabitants, 182 (17.7%) in a town with 100 000–499 999 inhabitants, 131 (12.7%) in a town with 500 000 inhabitants or more. Thirty-four respondents (3.3%) finished primary school, 31 (3%) finished secondary school, 222 (21.6%) finished vocational school, 360 (35%) finished high school, 39 (3.8%) were currently studying at university, 337 (32.7%) completed a university degree, 7 (0.7%) had other education. Five-hundred and nineteen (50.4%) respondents were married, 181 (17.6%) in informal relationship, 233 (22.6%) were not in a relationship, 25 (2.4%) widowed, 71 (6.9%) divorced. Five participants (0.5%) chose “other” as their relationship status. In terms of the cut-off criteria for personality impairment, 86 (8.3%) fell into the group of mild personality impairment risk, 33 (3.2%) into the group of moderate personality impairment risk, 17 (1.7%) into the group of severe personality impairment risk, 6 (0.6%) into the group of extremely severe personality impairment risk. One hundred sixty-five (16.0%) participants had the depressive symptoms’ score over 12, suggesting clinical depression risk.

### Analytic strategy

First, we performed Pearson’s r correlation analysis to explore the relationships between the measured variables. Next, all variables were standardized to *z*-scores (standardization on the whole sample). The focal analyses were performed with two-step clustering along with Kruskal-Wallis *H* test, chosen due to sample size imbalance across clusters. The two-step clustering approach has a crucial advantage over k-means and hierarchical clustering, as the number of clusters is defined automatically based on the Bayesian Information Criterion (BIC), and thus the final number of groups is not reliant on arbitrary decisions of a researcher^53^. The algorithm evaluates solutions with varying numbers of clusters and computes the change in BIC (ΔBIC) between consecutive models. The final solution was selected at the point where ΔBIC became negative for the last time, indicating that further increases in the number of clusters did not yield additional improvements in model fit but instead increased model complexity.

First, we performed the clustering solely on the two LPFS subscales: Self and Interpersonal Functioning (as indicated in our previous publication^41^. Then, we performed a focal analysis, and further clustering was done within the LPFS clusters, using the ICD-11 maladaptive personality traits. The standardization was not repeated after the first clustering, so that all results in the maladaptive personality traits are comparable between all subclusters. For all clusters we report the Silhouette metric, which refers to relative compactness and separability of clusters^53^. The values of the metric may take values from − 1 to 1, and the closer to 1, the more distinguishable and well apart are the clusters. Values lower than 0.25 indicate bad compactness and separability, 0.25 to 0.50 – a fair one, and over 0.50 – a good one^54,55^. Moreover, we reported proportion of group sizes, which shows the ratio of the largest to smallest cluster size. When describing the standardized results of the variables used for clustering, the following convention was used: result <−0.5; 0.5> – average, result <−1; − 0.5) – somewhat low, result (0.5; 1> - somewhat high, result less than − 1 – low, result higher than 1 – high.

The clusters were then compared in terms of the depressive symptoms. All *post hoc* tests were performed using Mann-Whitney *U* test with automatic Bonferroni correction accounting for multiple comparisons. The effects sizes were determined using Psychometrica.de calculation tool^56^, and further interpreted with guidance of Cohen^57^.

We expected to find subclusters of patterns of maladaptive personality traits within the clusters of self- and interpersonal functioning impairment levels. For exploratory purposes, we present the maladaptive personality domains’ clusters identified in the whole sample in Supplementary Material S1.

## Results

First, we present the correlation analysis showing the bivariate relationships between continuous variables (Table 1).

**Table 1:** Pearson’s r coefficients, means, standard deviations, skewness and kurtosis for study variables.

Variable	1	2	3	4	5	6	7	8	9
1. Negative Affectivity	-								
2. Disinhibition	0.54*	-							
3. Detachment	0.51*	0.44*	-						
4. Dissociality	0.34*	0.57*	0.35*	-					
5. Anankastia	0.18*	− 0.27*	0.16*	− 0.03*	-				
6. LPFS-Self	0.72*	0.54*	0.52*	0.34*	0.01	-			
7. LPFS-Interpersonal	0.61*	0.50*	0.54*	0.43*	0.08*	0.68*	-		
8. LPFS-General score	0.73*	0.57*	0.57*	0.42*	0.05	0.93*	0.90*	-	
9. Depressive symptoms	0.68*	0.46*	0.44*	0.25*	0.03	0.70*	0.52*	0.68*	-
M	2.94	2.13	2.54	2.06	3.40	2.00	2.01	2.01	6.79
SD	0.75	0.62	0.68	0.53	0.49	0.77	0.62	0.64	5.82
Minimum	1.00	1.00	1.00	1.00	1.00	1.00	1.00	1.00	0.00
Maximum	5.00	4.75	4.58	4.08	5.00	4.00	4.00	3.92	27.00
Skewness	− 0.04	0.43	0.08	0.41	− 0.58	0.43	0.27	0.28	1.05
Kurtosis	− 0.21	0.04	− 0.38	− 0.03	2.31	− 0.68	− 0.25	− 0.55	0.67

*p <.001.

Data from Table 1 showed that there are consistently positive and significant correlations between four out of five maladaptive personality traits: Negative Affectivity, Disinhibition, Detachment and Dissociality (correlation coefficients ranging from *r* =.34 to *r* =.57; *p* <.001). Anankastia also showed significant and positive relationships with Negative Affectivity (*r* =.18; *p* <.001) and Detachment (*r* =.16; *p* <.001), however, significant and negative ones with Disinhibition (*r* = −.27; *p* <.001) and Dissociality (nevertheless, the latter relation was very weak: *r* = −.03; *p* <.001).

Impairments in Self, Interpersonal and General Personality Functioning related significantly and positively to Negative Affectivity (correlation coefficients ranging from *r* =.61 to *r* =.73; *p* <.001), Disinhibition (correlation coefficients ranging from *r* =.50 to *r* =.57; *p* <.001), Detachment (correlation coefficients ranging from *r* =.52 to *r* =.57; *p* <.001), and Dissociality (correlation coefficients ranging from *r* =.34 to *r* =.43; *p* <.001). Self and General Personality Functioning impairments were not significantly associated with Anankastia. Only Interpersonal Functioning impairment related significantly, positively and very weakly to this trait (*r* =.08; *p* <.001). Moreover, depressive symptoms related significantly and positively to Negative Affectivity (*r* =.68; *p* <.001), Disinhibition (*r* =.46; *p* <.001), Detachment (*r* =.44; *p* <.001) and Dissociality (*r* =.25; *p* <.001), but were not significantly associated with Anankastia.

### First cluster analysis: LPF profiles and comparisons

The first cluster analysis focused on the LPFS subscales: Self and Interpersonal Functioning, and has been already published^41^. Standardized results in these subscales were introduced to the clustering procedure as quantitative variables. We present the means and standard deviations of the variables used for clustering across the three clusters, along with the importance of each of the two subscales in Supplementary Material S2.

**Fig. 1:**
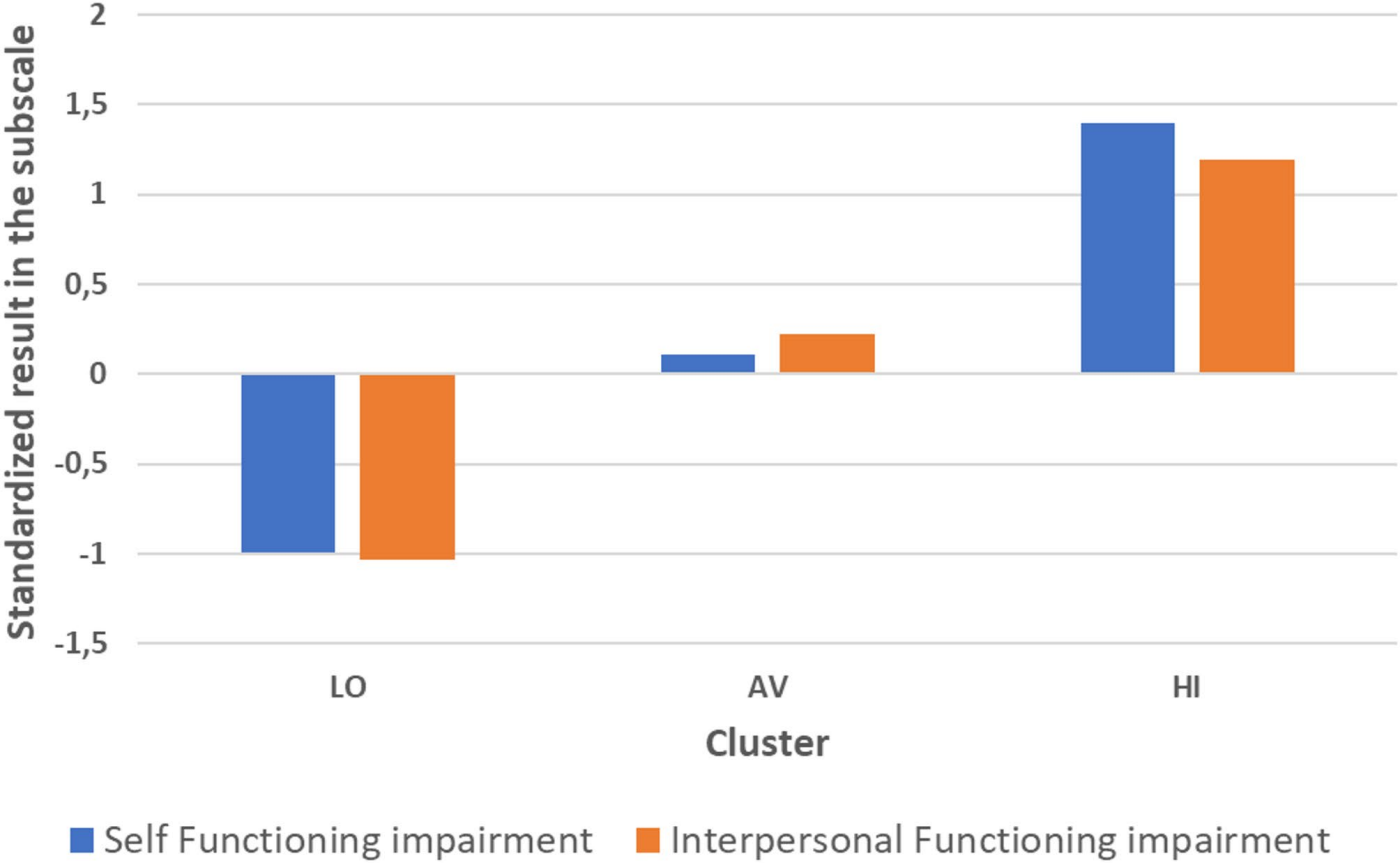
Results in the two subscales across three distinguished LPFS clusters. Note: LO – Low Impairment cluster, AV – Average Impairment cluster, HI – High Impairment cluster.Results in the two subscales across three distinguished LPFS clusters. Note: LO – Low Impairment cluster, AV – Average Impairment cluster, HI – High Impairment cluster.

Figure 1 shows a visualization of the revealed clusters of LPFS.

Cluster 1 was named Low Impairment (LO), given the somewhat low level of impairment in self-functioning and interpersonal functioning. It consisted of *N* = 347 (33.7%) participants. None of them met a cut-off score for personality impairment (range of LPFS general score: 12–20). Cluster 2 was named Average Impairment (AV), given the average level of impairment in both intra- and interpersonal functioning. It consisted of *N* = 471 (45.7%) participants. None of them met a cut-off score for personality impairment (range of LPFS general score: 20–30). Cluster 3 was named High Impairment (HI), given the high level of dysfunction in both intra- and interpersonal functioning. It consisted of *N* = 212 (20.6%) participants. The range of LPFS general score in this cluster was 31–47. One hundred forty-two (67.0%) met a cut-off score for personality impairment.

We controlled whether the clusters differ significantly in terms of the results in LPFS subscales. Results of a Kruskal-Wallis *H* test and a subsequent *U* Mann-Whitney *post hoc* test indicated that in case of both subscales, all clusters differed significantly between each other with *p* <.001. Regarding the demographic characteristics of the clusters, as reported in our previous paper (for more details see^41^, in cluster LO there were significantly older participants than in AV and HI, and in AV there were significantly older participants than in HI. The proportion of women was higher in HI cluster than in LO cluster. There were no significant differences in terms of size of place of residence between the members of each of the clusters. In terms of the last finished education level, LO cluster had more people with vocational education compared to AV and HI, while HI had the highest share of individuals who had a higher education degree. For the relationship status, HI had the largest proportion of single individuals, followed by group AV, with group LO having the fewest. Conversely, married individuals are most common in group LO, less so in group AV, and least in group HI. The three revealed LPFS clusters were used in the further analysis as the grouping variable in order to disentangle the maladaptive personality domains’ profiles embedded inside the low, average and high personality functioning impairment functioning.

### Second cluster analysis: subclusters of maladaptive personality traits within clusters of LPFS

Next, we performed a two-step cluster analysis of the maladaptive personality traits across the three previously distinguished LPFS clusters in the community sample. This analysis has not been published elsewhere. The standardized results of the maladaptive personality traits questionnaire were introduced as quantitative variables to the analysis.

In the case of the low personality functioning impairment structure cluster, two subclusters of maladaptive personality traits were revealed, Silhouette = 0.40, proportion of group sizes = 1.20. The first group consisted of 189 participants (54.5%), BIC = 766.38, and the second of 158 participants (45.5%), ΔBIC = −208.18.

In the case of average personality functioning impairment cluster, three subclusters of maladaptive personality traits were revealed, Silhouette = 0.30, proportion of group sizes = 1.11. The first group consisted of 161 participants (34.2%), BIC = 791.05, the second of 147 participants (31.2%), ΔBIC = −186.87, and the third of 163 participants (34.6%), ΔBIC = −97.91.

In the case of high personality functioning impairment cluster, four subclusters of maladaptive personality traits were revealed, Silhouette = 0.30, proportion of group sizes = 1.89. The first group consisted of 51 participants (24.1%), BIC = 731.61, the second of 60 participants (28.3%), ΔBIC = −61.14, the third of 66 participants (31.1%), ΔBIC = −16.92, and the fourth of 35 participants (16.5%), ΔBIC = −5.96.

Table 2 presents the means and standard deviations, as well as importance of maladaptive personality traits across the LPFS clusters and the maladaptive personality traits subclusters inside them.

**Table 2:** Means, standard deviations, and importance of maladaptive personality traits (MPT) across the subclusters.

LPFS cluster	MPT subcluster	Negative Affectivity		Disinhibition		Detachment		Dissociality		Anankastia	
		*M (SD)*	Importance	*M (SD)*	Importance	*M (SD)*	Importance	*M (SD)*	Importance	*M (SD)*	Importance
Low	1	− 0.41 (0.65)	0.42	− 0.09 (0.64)	1.00	− 0.22 (0.75)	0.50	− 0.04 (0.65)	0.70	− 0.12 (0.77)	0.01
	2	−1.24 (0.74)		−1.26 (0.42)		−1.18 (0.69)		−1.05 (0.57)		0.05 (1.40)	
Average	1	− 0.28 (0.59)	0.27	− 0.21 (0.52)	1.00	− 0.50 (0.56)	0.62	− 0.17 (0.78)	0.49	− 0.21 (0.78)	0.65
	2	0.37 (0.82)		− 0.44 (0.66)		0.53 (0.77)		− 0.29 (0.72)		0.82 (0.70)	
	3	0.37 (0.60)		0.88 (0.54)		0.49 (0.61)		0.78 (0.74)		− 0.44 (0.65)	
High	1	1.43 (0.58)	0.45	1.15 (1.04)	0.86	1.51 (0.62)	0.51	1.21 (0.97)	0.61	0.67 (0.57)	1.00
	2	1.01 (0.59)		1.67 (0.62)		0.65 (0.83)		1.13 (1.08)		−1.01 (0.73)	
	3	0.38 (0.63)		0.41 (0.53)		0.02 (0.74)		0.22 (0.73)		− 0.22 (0.72)	
	4	1.35 (0.72)		− 0.43 (0.59)		1.00 (0.88)		− 0.81 (0.67)		1.12 (0.72)	

Importance describes the impact a particular variable has on determining clusters, relative to the variable that has most impact (with importance equal to 1.00.

**Fig. 2:**
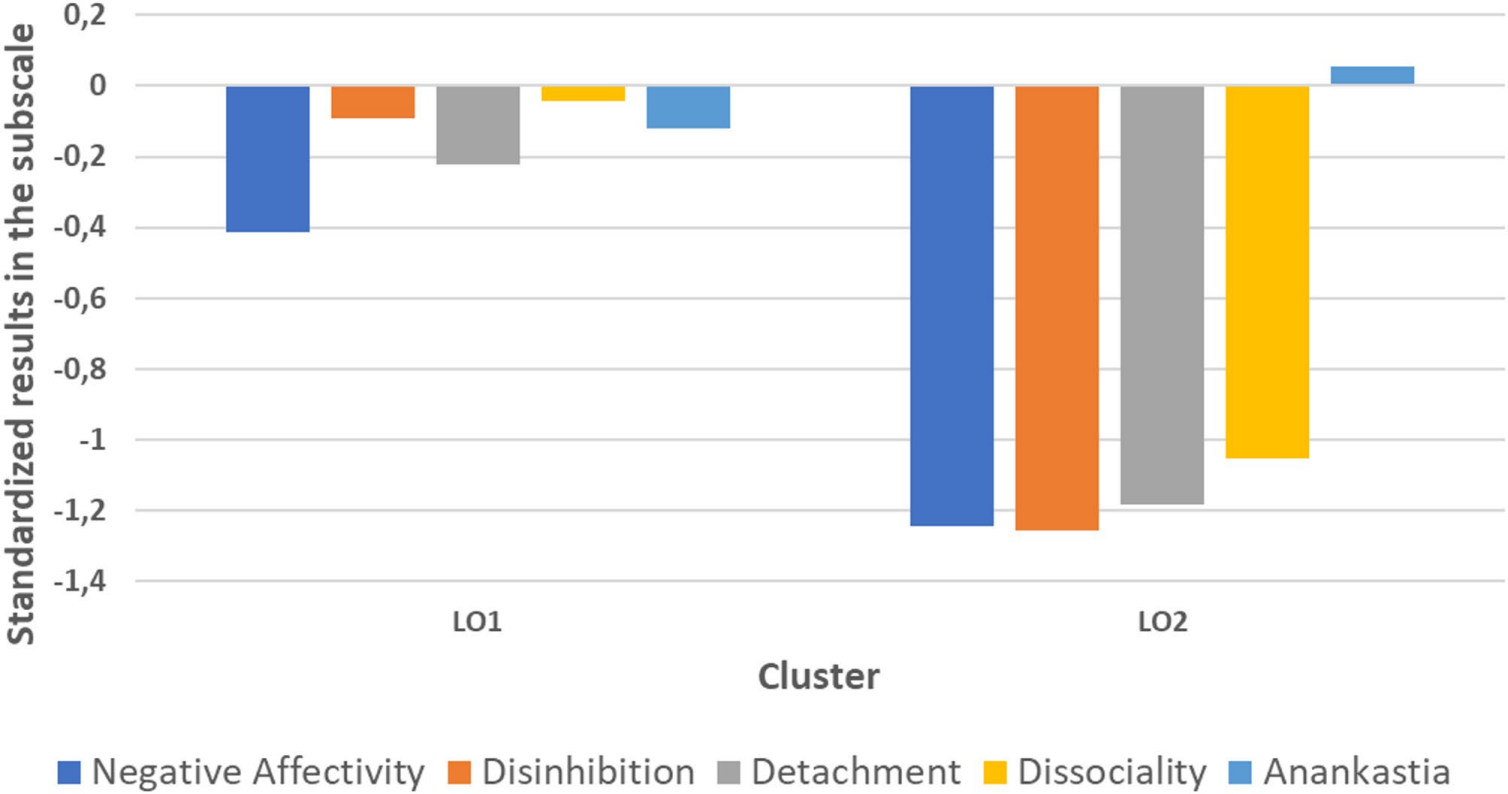
Maladaptive personality traits’ clusters within the low personality functioning impairment (abbreviation: LO) cluster. Numbers denote the subsequent clusters that emerged.

Within the LO impairment LPFS cluster (characterized by a low level of self- and interpersonal-functioning dysfunction in the community sample), the LO1 cluster was characterized by an average level across all maladaptive personality domains. A LO2 cluster was characterized by low levels of all maladaptive personality domains excluding an average result in Anankastia. We controlled whether these subclusters differ significantly in terms of the results in each of the maladaptive personality trait. Results of a *U* Mann-Whitney’s test revealed that the clusters differed significantly in the case of all traits (*p* <.001 except for Anankastia with *p* <.01). Figure 2 shows a visualization of the clusters of maladaptive personality traits in groups of low LPFS, based on data from Table 2.

Within the AV impairment LPFS cluster (with an average level of self and interpersonal functioning dysfunctions in the community sample), an AV1 cluster was characterized by an average level of all maladaptive personality domains. An AV2 cluster was characterized by average levels of Negative Affectivity, Disinhibition and Dissociality, and somewhat high levels of Detachment and Anankastia. An AV3 cluster was characterized by average levels of Negative Affectivity, Detachment and Anankastia, and somewhat high levels of Disinhibition and Dissociality.

**Fig. 3:**
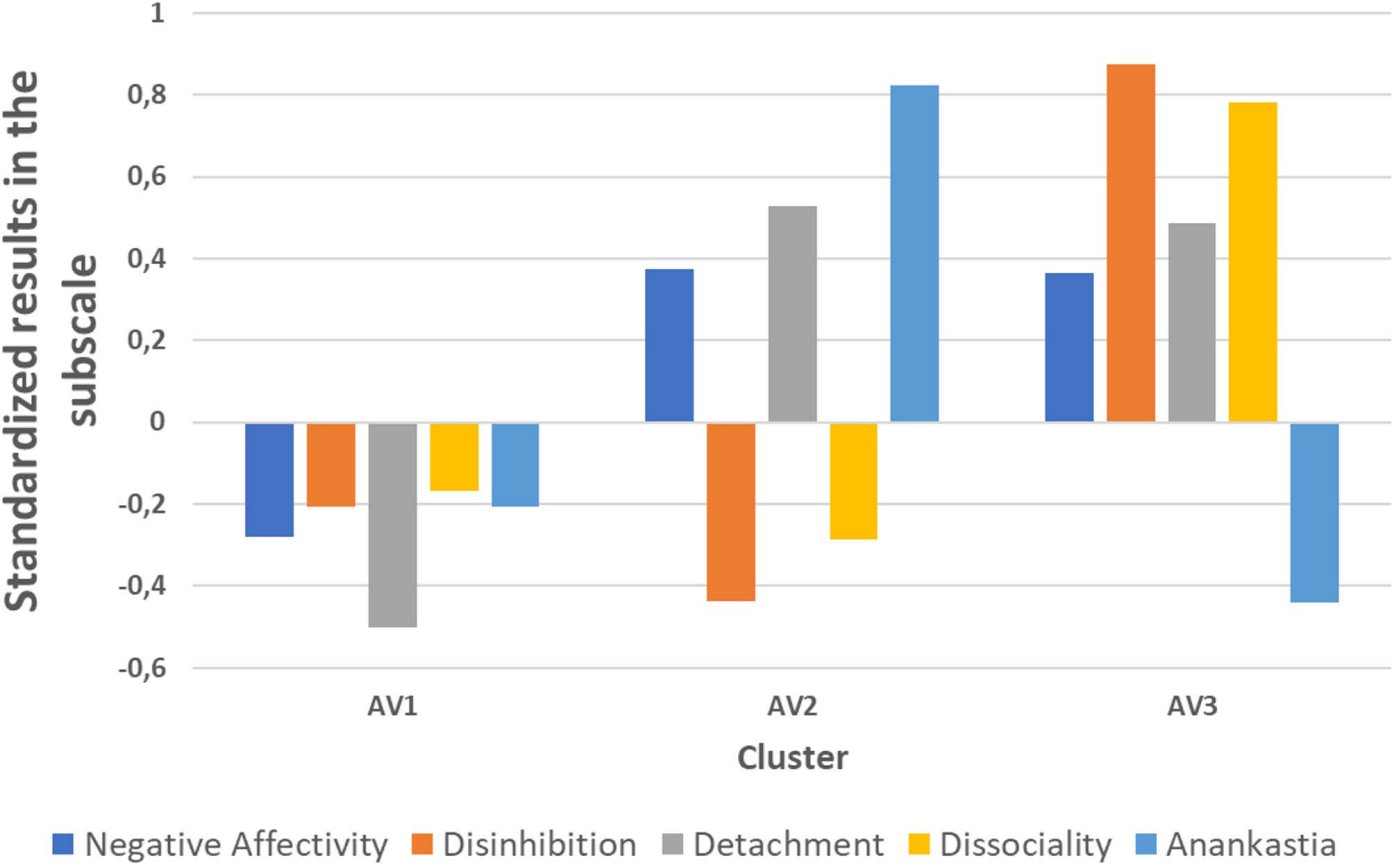
Maladaptive personality traits’ clusters within the average personality functioning impairment (abbreviation: AV) cluster. Numbers denote the subsequent clusters that emerged.

Results of a Kruskal-Wallis’ *H* and further *post hoc* Mann-Whitney’s *U* tests revealed that the clusters differed in the case of all traits on *p* <.001, except for the difference on Disinhibition between Subcluster 1 and 2 which was on *p* <.05 and Anankastia between Subcluster 1 and 3, which was on *p* <.01; and three cases in which the subclusters did not differ: Subclusters 1 and 2 in terms of Dissociality (*p* =.653) and Subclusters 2 and 3 in terms of Negative Affectivity (*p* = 1.00), and Detachment (*p* = 1.00). Figure 3 shows a visualization of the clusters of maladaptive personality traits in groups of average LPFS, based on data from Table 2.

In the high LPFS cluster (with the highest level of self and interpersonal functioning dysfunctions in the community sample), HI1 cluster was characterized by high levels of all maladaptive personality domains except Anankastia, which was only somewhat high. HI2 cluster was characterized by high Negative Affectivity, Disinhibition and Dissociality, as well as somewhat high level of Dissociality and low level of Anankastia. HI3 cluster was characterized by average levels of all maladaptive personality traits. HI4 cluster was characterized by high levels of Negative Affectivity and Anankastia, somewhat high level of Detachment, average level of Disinhibition and somewhat low level of Dissociality.

**Fig. 4:**
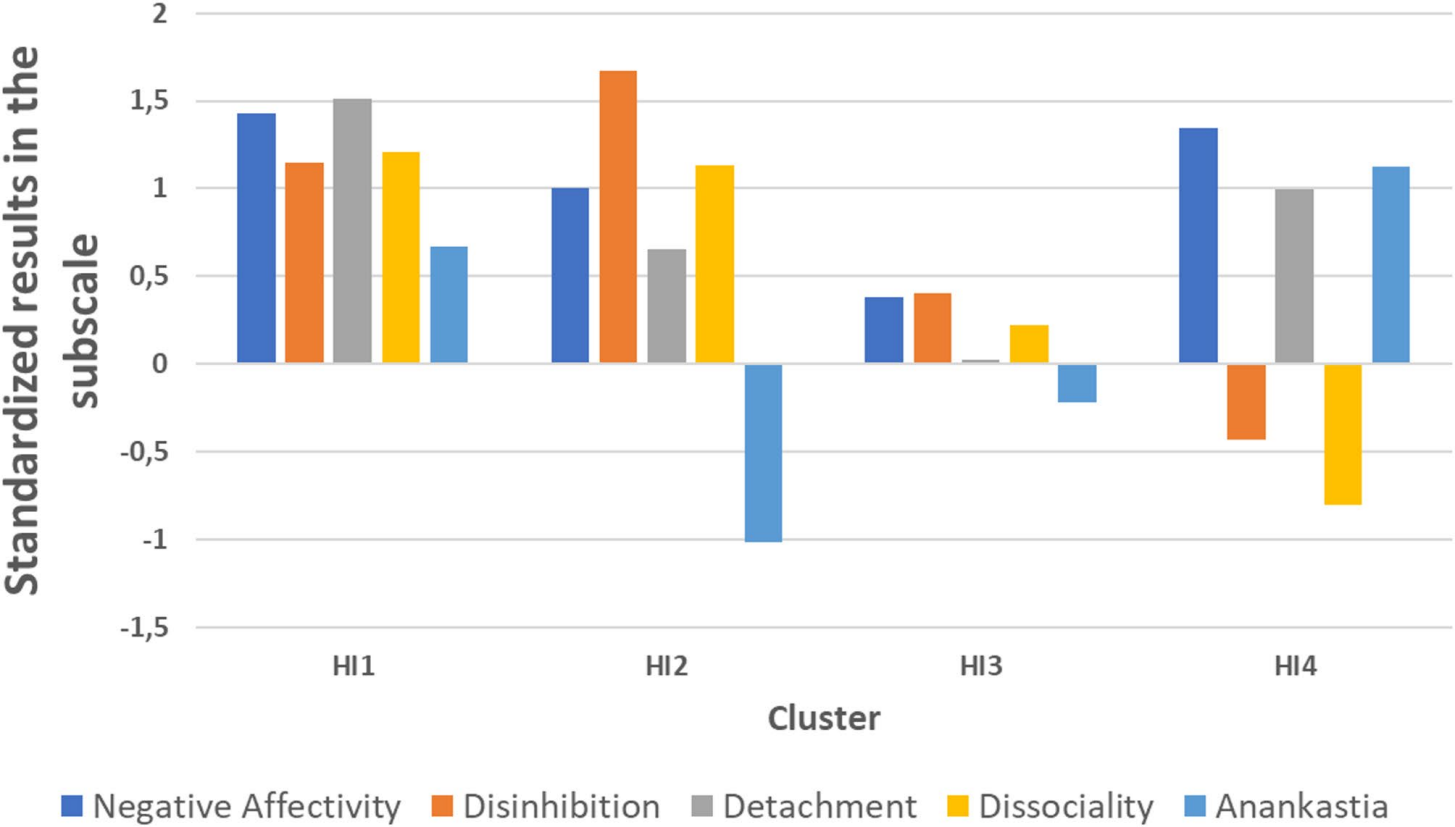
Maladaptive personality traits’ clusters within the high personality functioning impairment (abbreviation: HI) cluster. Numbers denote the subsequent clusters that emerged.

Results of a Kruskal-Wallis’ *H* and further *post hoc* Mann-Whitney’s *U* tests revealed that the clusters, with exceptions stated below, differed in the case of all traits on *p* <.001, except for the differences between Subclusters 1 and 2 in terms of Negative Affectivity and Disinhibition (*p* <.05), between Subclusters 2 and 3 in terms of Detachment (*p* <.01), and Subclusters 3 and 4 in terms of Disinhibition (*p* <.01). The exceptions in which the traits did not differ across subclusters were: lack of differences between Subcluster 1 and 4 in terms of Negative Affectivity (*p* = 1.00), Detachment (*p* =.077), and Anankastia (*p* =.512); lack of differences between Subcluster 2 and 4 in terms of Negative Affectivity (*p* =.230) and Detachment (*p* =.364), and between Subcluster 1 and 2 in terms of Dissociality (*p* = 1.00). Figure 4 shows a visualization of the clusters of maladaptive personality traits in groups of high LPFS, based on data from Table 2.

### Differences in depressive symptoms’ intensity across subclusters

Next, we performed a Kruskal-Wallis’ *H* test to assess the differences between the nine subclusters in terms of the depressive symptoms (PHQ sum score). The test proved statistically significant, *H*(8) = 418.49; *p* <.001, suggesting the existence of at least one intergroup difference between subclusters. Therefore, a *post hoc U*-Mann-Whitney test was performed to find these differences. Supplementary Material Table S3 shows the PHQ mean ranks in nine subclusters.

The highest mean rank of depressive symptoms was observed for subcluster HI1, and the lowest for LO2 subcluster. Supplementary Material Table S4 presents the results of the *U* Mann-Whitney’s tests.

Results show that all pairs of subclusters differed significantly (*p*-corrected < 0.05) in terms of the depressive symptoms intensity, except for pairs: AV1-AV2, AV2-AV3, AV2-HI3, AV3-HI3, HI3-HI2, HI3-HI4, HI3-HI4, HI2-HI4, HI2-HI1, HI4-HI1. All effect sizes were of a large size according to conventions by Cohen^57^ transformation performed through algorithms by Lenhard & Lenhard^56^.

## Discussion

The obtained results partially supported our hypothesis (H1), as we observed subclusters of maladaptive personality traits within the clusters of general personality functioning impairment, and some of them differed in their levels of depressive symptoms. Nevertheless, higher depressive symptomatology was primarily observed in clusters exhibiting relatively high Negative Affectivity trait, and it appeared on all levels of personality impairment. First, we will characterize the obtained subclusters to provide context for the interpretations of the obtained differences in depressive symptoms.

We found that the higher the level of personality functioning impairment, the more subclusters emerged, each characterized by higher levels of maladaptive personality domains. This may suggest that maladaptive trait measures differentiate personality profiles better among people with more pronounced personality functioning. Such differentiation is consistent with the conceptual frameworks of ICD-11 and DSM-5^58^.

One general pattern was observed across all levels of personality functioning impairment: the presence of a subcluster characterized by average levels of all five maladaptive domains (LO1, AV1, HI3). Nevertheless, these profiles differed significantly in terms of the severity of depressive symptoms. This suggests that the level of impairment in personality functioning that differentiates these particular “average” profiles in which none of the traits predominates over the others may be related to depressive severity beyond maladaptive traits. This finding provides a cue for clinical practice and for further research on the link between personality functioning and depression.

At the low level of personality functioning impairment, we observed two subclusters. One (LO1) displayed an average level of all five maladaptive personality domains, indicating a balanced profile. The second (LO2) exhibited low levels across traits except for average Anankastia. This finding adds to the ongoing discussion regarding what constitutes a normal or adequate intensity of pathological traits. The LO1 profile displayed average levels across all five pathological trait domains, reflecting, for example, mild emotional instability or stress sensitivity (Negative Affectivity), slight social withdrawal or limited interpersonal engagement (Detachment), occasional self-centeredness or difficulties in assertive interactions (Dissociality), minor impulsivity or challenges with delaying gratification (Disinhibition), and subtle perfectionistic tendencies or rigidity (Anankastia)—without evidence of severe dysfunction. This presentation is consistent with the ICD-11 classification of “no PD” or “personality difficulty.” LO1 likely represents subclinical personality traits that may influence emotional and interpersonal functioning in everyday life without meeting criteria for a formal disorder.

It is possible that participants in the LO2 subcluster represent a generally well-adjusted group, with the exception of Anankastia. This exception may be related to the recruitment process, as participants were members of a panel in which they earned money by completing surveys. Given that maladaptive personality domains resemble the Big Five traits^11^, the presence of average Anankastia might reflect conscientiousness, which is necessary for engaging in sustained activities with delayed rewards, such as survey completion. Interestingly, this particular subcluster was not observed at higher levels of personality functioning impairment. This suggests that personality dysfunction is consistently associated with at least average levels of trait pathology, indicating a link between impairment in personality functioning and the manifestation of maladaptive personality domains.

At the average level of personality functioning impairment, three subclusters were identified. AV1 displayed all stylistic traits at an average level. AV2 also showed traits at an average level, with somewhat higher Detachment and Anankastia. AV3 presented traits at an average level, with somewhat higher Disinhibition and Dissociality, and average but slightly lower Anankastia.

At the high level of personality functioning impairment, four subclusters emerged. The first (HI1) exhibited all traits at high levels, with slightly elevated Anankastia. This cluster appears to represent the most severe psychopathological profile. As Bastiaens et al.^59^ demonstrated, personality models based on maladaptive personality traits can identify clusters characterized by simultaneously high Disinhibition and Anankastia, similar to our HI1 subcluster. HI1 also displayed the highest levels of depressive symptoms and was the only high-impairment cluster that did not demonstrate a clear Disinhibition/Anankastia opposition. Taken together with the overall elevation of traits, this suggests that HI1 may represent individuals with more than one PD. Previous studies have shown that such mixed psychopathology is common among clinically depressed patients^60^ and may indicate a complex (diffuse) PD^61^.

HI2 was characterized by high Negative Affectivity, Disinhibition, and Dissociality, with somewhat higher Detachment and somewhat lower Anankastia. Visually, this cluster resembled a more severe form of AV3, suggesting that increasing levels of personality dysfunction are associated with more pronounced maladaptive domains. Interestingly, both AV3 and HI2 exhibited very low Anankastia in combination with high Disinhibition. Disinhibition and Anankastia are conceptualized as opposite poles of the same bipolar dimension, merged in the four-factor model and separated in the five-factor model^48,49^. In our study, this Disinhibition/Anankastia opposition was evident in six out of nine subclusters.

HI3 displayed all stylistic traits at an average level. HI4 exhibited high Negative Affectivity and Anankastia, somewhat high Detachment, average Disinhibition, and somewhat low Dissociality. Visually, this cluster resembled a more severe form of AV2. It further highlighted how greater impairment in personality functioning corresponds to stronger expression of maladaptive traits.

The maladaptive trait subclusters identified within the levels of personality functioning impairment show some parallels with well-described personality configurations in AMPD-informed research (thus, a framework similar but not identical to the used ICD-11-based model), particularly the resilient, undercontrolled and overcontrolled types^62,63^. The resilient profile is characterized by relatively balanced and low-to-average levels across all maladaptive trait domains, suggesting adequate emotional regulation, impulse control, and interpersonal functioning. This profile corresponds most closely to the LO1 subcluster, which shows no pronounced elevations in any pathological trait domain. The undercontrolled profile is marked by elevated Disinhibition, Dissociality and Negative Affectivity. In the present analysis, this configuration is most evident in the AV3 subcluster and its more severe equivalent, HI2, both of which display high Disinhibition and low Anankastia. The overcontrolled profile is primarily defined by heightened Negative Affectivity and Anankastia, with comparatively lower Disinhibition. This configuration is most closely approximated by the HI4 subcluster. Altogether in both theoretical frameworks the level of personality functioning impairment differentiates the clinical relevance and depressive burden associated with otherwise similar maladaptive trait profiles.

In line with H1, we found that the severity of depressive symptoms increases significantly with higher levels of personality pathology. Nevertheless, subclusters in which Negative Affectivity was on a relatively high level, had depressive symptoms most pronounced. It is in line with previous data^28^ and the characteristics of this maladaptive trait^6^.

At the subcluster level, those within the same degree of personality functioning impairment generally did not differ, with only a few exceptions. Notably, subclusters at the low level of impairment showed differences in depressive symptoms, with LO2, the generally well-adjusted group, exhibiting exceptionally low rates. This aligns with previous studies confirming associations between the level of personality dysfunction^16^ and pathological personality traits^64^ with depression. Personality pathology may increase vulnerability to developing depression, contribute to more severe symptomatology, and raise the likelihood of chronic or recurring episodes^65–67^.

Additionally, no significant differences in depressive symptoms were found between AV1 and AV2. This may be attributed to the average level of Negative Affectivity observed in both subclusters, which is the most predictive of depressive symptomatology out of all maladaptive personality traits^28^.

Another noteworthy finding emerged in HI3, the subcluster characterized by average levels of maladaptive personality domains within the high personality-functioning impairment cluster. Although no differences in depressive symptoms’ severity were observed between HI3 and AV2 or AV3, it differed significantly in these terms from HI1. This suggests that individuals in HI3 exhibited substantially lower levels of depressive symptoms compared to those in the high personality psychopathology group (HI1). This supports the idea that a more balanced presentation of maladaptive traits may contribute to greater stability and, consequently, lower depressive symptomatology. Thus, HI3 represents a subgroup that, despite experiencing impairments in personality functioning, demonstrates depressive symptoms more similar to those of individuals with average levels of personality dysfunction.

In sum, greater severity of personality impairment is associated with more distinct subclusters, suggesting that the expression of pathological traits differs according to the overall level of dysfunction. Negative Affectivity is the most prominent factor that correlates with depressive symptomatology across the subclusters. These findings extend existing knowledge by demonstrating that levels of personality functioning not only index the degree of dysfunction but may also help predict the variability and manifestation of pathological traits across individuals.

### Clinical implications

Our results emphasize the crucial role of personality functioning in the treatment of depression. In our study, more severe personality impairments were associated not only with a greater variety of maladaptive personality profiles but also with more severe depressive symptoms. These impairments often contribute to social difficulties, which can increase social withdrawal and relatedness frustration, potentially exacerbating depressive symptomatology^68,69^. Over time, this can trigger maladaptive behavioral patterns and further intensify personality problems.

Our findings regarding significant positive correlations between depressive symptoms and Negative Affectivity, Disinhibition, Detachment, and Dissociality highlight the importance of screening for personality pathology among patients with depression^16,70,71^. Maladaptive personality traits’ screening enables treatment that takes into account both personality traits and the severity of personality dysfunction. If these traits are ignored, patients may experience additional suffering due to inadequate psychotherapeutic interventions. Nevertheless, our study confirms the central role of Negative Affectivity in the severity of depressive symptomatology regardless of the severity of personality impairment, suggesting that patients displaying this trait should be specifically observed in terms of depressive risk.

Subclusters with balanced or average trait configurations (LO1, AV1, HI3) showed substantially lower depressive symptomatology even at higher levels of personality functioning impairment. From a clinical perspective, this suggests that treatment planning for depression may benefit from attending not only to the presence of affective distress but also to the broader pattern of impulse control, interpersonal functioning, and rigidity versus flexibility, as it may help in choosing different therapeutic approaches and interventions.

This also underscores the importance of close collaboration between psychiatrists and psychotherapists, as well as the integration of different therapeutic modalities. A psychoanalytic/psychodynamic understanding of personality pathology (as reflected in both ICD-11 and DSM-5) is essential, for example, when applying cognitive-behavioral techniques, since the effectiveness of such interventions may depend on the specific level of personality functioning: low (LO), average (AV), or high (HI), as observed in our study^72^. In other words, understanding a patient’s underlying personality structure can guide the selection and adaptation of both psychoanalytically driven and cognitive-behaviorally driven methods, ensuring that treatment addresses not only depressive symptoms but also the personality patterns that contribute to them^73^.

By taking personality into account, clinicians can make more informed decisions regarding the timing and type of pharmacotherapy. A personalized, integrative approach that combines pharmacological interventions with psychotherapeutically informed strategies can optimize outcomes by simultaneously targeting depressive symptoms and underlying personality vulnerabilities.

### Limitations and future research directions

Our study, conducted on a representative sample of Polish adults, offers valuable insights into psychopathological patterns in a diverse population. However, several limitations must be acknowledged. Firstly, the study design was cross-sectional, which prevents us from establishing causal relationships between variables. It is possible that some participants, particularly those exhibiting the highest levels of depressive symptoms, presented with “state-dependent personality pathology”^74^ rather than a trait-based, persistent PD. Intervention studies are needed to determine the extent to which these personality dysfunctions remain stable following treatment of depressive symptoms.

Moreover, in the current study, no additional data were collected that could determine whether the cluster patterns would replicate in a different sample or at another point in time. Nevertheless, we took steps to ensure representativeness in terms of key demographic characteristics (gender, age, size of place of residence, education), thereby attempting to make our analyses generalizable at the cross-sectional level. Future studies, particularly longitudinal designs, such as those employing experience sampling methods, could explore how individuals with specific personality pathologies perceive daily events and how this relates to depressive symptoms.

Another limitation concerns our reliance on self-report measures administered online, which cannot fully substitute for clinical diagnoses. Clinician assessments allow face-to-face interactions, enable monitoring and control of study conditions (e.g., environment, participant concentration), and provide opportunities for follow-up questions and non-verbal communication, an essential element of assessment^75^ that is inevitably absent in large-scale online surveys. Accordingly, our measurement of clinical constructs should be considered screening, not diagnosis. Moreover, because our sample consisted of non-clinical individuals, the generalizability of findings to clinical populations may be limited.

Additionally, the number of clusters was defined automatically by the employed algorithm on a randomly sorted database to best reflect the sample characteristics. However, alternative clustering approaches might yield different insights. We also applied the LPFS, designed for DSM-5, together with the ICD-11 maladaptive personality traits measure. This choice was guided by the psychometric robustness of the Polish adaptations and our aim to include Anankastia in the models (which is not possible when using DSM-5-based measures). Still, this reflects a limitation in that we inferred from two related but distinct theoretical frameworks of PDs.

Lastly, although the PHQ-9 provides simple and accessible items, it is primarily a screening tool rather than a comprehensive psychiatric interview. Future research should investigate the associations between difficulties in personality functioning and a broader spectrum of psychopathological symptoms beyond depression. It would also be valuable to broaden the scope of psychiatric research on PDs and depression by considering the biological underpinnings of mental health.

## Supplementary Information

Below is the link to the electronic supplementary material.

Supplementary Material 1

## Data Availability

The project is based on data published at https://osf.io/5seuv/. The full list of measures used in the whole study is available in the Supplementary Material S5. The research question and analysis reported here is a secondary, *ex definitio* exploratory analysis. No experimental manipulations were employed in the study. Observations of inattentive respondents (namely, those who did not answer correctly to at least one of three attention check questions) were excluded from further analysis. Other exclusion criteria were not applied.
