# Supplementary material for: Variations in maladaptive personality patterns and depressive symptoms’ severity across personality functioning profiles in a community sample
Source: Sci Rep. 2026 Apr 24;16:18887. doi: 10.1038/s41598-026-50285-9 (PMC13276178; doi:10.1038/s41598-026-50285-9)
Supplement: Supplementary file 1 — Supplementary Material 1 [file 41598_2026_50285_MOESM1_ESM.docx]

**Supplementary Material**

**Supplementary Material S1**

**Clusters of maladaptive personality domain patterns across the whole sample**

The analysis revealed two clusters, Silhouette = .40, proportion of group sizes = 1.15. The first cluster was constituted of 479 participants (46.5%); BIC = 3636.58, the second one of 551 participants (53.5%); ΔBIC = -683.95. We present the means and standard deviations of the variables used for clustering across the three clusters, along with the importance of each of the five subscales.

Table S1 *Means, standard deviations, and importance of ICD-11 maladaptive personality domains across the subclusters*

| **Cluster** | **Negative Affectivity** | | **Disinhibition** | | **Detachment** | | **Dissociality** | | **Anankastia** | |
| --- | --- | --- | --- | --- | --- | --- | --- | --- | --- | --- |
|  | ***M (SD)*** | **Importance** | ***M (SD)*** | **Importance** | ***M (SD)*** | **Importance** | ***M (SD)*** | **Importance** | ***M (SD)*** | **Importance** |
| 1 | .71  (.71) | 1.00 | .68  (.87) | .91 | .66  (.76) | .84 | .56  (.95) | .56 | .01  (.94) | .00 |
| 2 | -.61 (.79) |  | -.59 (.68) |  | -.57  (.82) |  | -.48 (.76) |  | -.01 (1.05) |  |

*Note.* Importance describes the impact a particular variable has on determining clusters, relative to the variable that has most impact (with importance equal to 1.00)


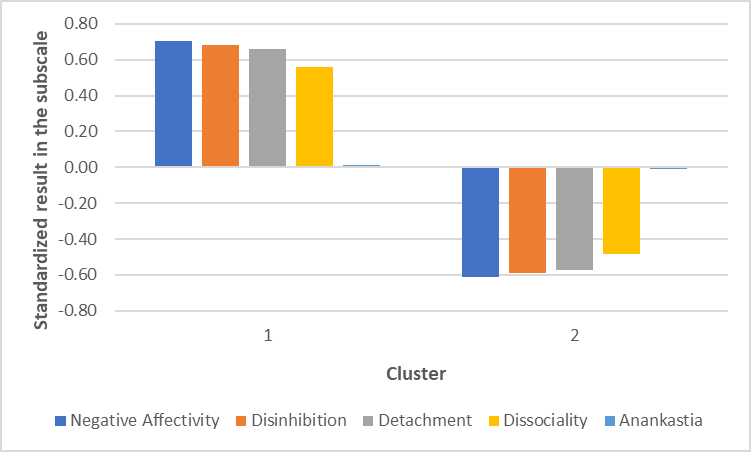


Figure S1. *Results in the two subscales across two distinguished ICD-11 clusters*

Cluster 1 was characterized by relatively high levels of maladaptive personality domains, except for an average level of Anankastia. Cluster 2 was characterized by relatively low levels of maladaptive personality domains, except for an average level of Anankastia.

We controlled whether the clusters differ significantly in terms of the results in ICD-11 subscales. Results of a *t*-test indicated that in case of all subscales, both clusters differed significantly between each other with *p* < .001 except for Anankastia.

**Supplementary Material S2**

**Means, standard deviations, and importance of LPFS subscales in clusters**

Table S2 *Means, standard deviations, and importance of LPFS subscales in clusters*

| **Cluster** | **Self Functioning (impairment)** | | **Interpersonal Functioning (impairment)** | |
| --- | --- | --- | --- | --- |
|  | ***M (SD)*** | **Importance** | ***M (SD)*** | **Importance** |
| 1 | -.99 (.35) | 1.00 | -1.03 (.48) | .85 |
| 2 | .11 (.59) |  | .22 (.55) |  |
| 3 | 1.39 (.55) |  | 1.19 (.72) |  |

*Note.* Importance describes the impact a particular variable has on determining clusters, relative to the variable that has most impact (with importance equal to 1.00)

**Supplementary Material S3**

**Mean ranks of depressive symptoms in nine subclusters**

Table S3 *Mean ranks of depressive symptoms in nine subclusters*

| **Subcluster** | ***N*** | **Mean rank** |
| --- | --- | --- |
| LO1 | 189 | 356.92 |
| LO2 | 158 | 232.13 |
| AV1 | 161 | 477.00 |
| AV2 | 147 | 576.29 |
| AV3 | 163 | 597.54 |
| HI1 | 51 | 891.26 |
| HI2 | 60 | 805.73 |
| HI3 | 66 | 684.44 |
| HI4 | 35 | 827.14 |

*Note.* LO – Low Impairment cluster, AV – Average Impairment cluster, HI – High Impairment cluster. Numbers reflect the number of a specific cluster, as described in the manuscript main body.

**Supplementary Material S4**

**Results of Mann-Whitney’s *U* subcluster comparison in terms of depressive symptoms’ severity**

Table S4 *Results of Mann-Whitney’s U subcluster comparison in terms of depressive symptoms’ severity*

| **Compared samples** | ***U*** | ***SE*** | ***z*** | ***p*** | ***p-corrected*** | ***η^2^*** |
| --- | --- | --- | --- | --- | --- | --- |
| LO2-LO1 | 124.79 | 31.99 | 3.90 | <.001 | .003 | .73 |
| LO2-AV1 | -244.88 | 33.23 | -7.37 | <.001 | .000 | .78 |
| LO2-AV2 | -344.16 | 34.01 | -10.12 | .000 | .000 | .79 |
| LO2-AV3 | -365.42 | 33.13 | -11.30 | .000 | .000 | .79 |
| LO2-HI3 | -452.31 | 43.50 | -10.40 | .000 | .000 | .73 |
| LO2-HI2 | -573.60 | 45.00 | -12.75 | .000 | .000 | .75 |
| LO2-HI4 | -595.02 | 55.44 | -10.73 | .000 | .000 | .65 |
| LO2-HI1 | -659.14 | 47.80 | -13.79 | .000 | .000 | .75 |
| LO1-AV1 | -120.09 | 31.83 | -3.77 | <.001 | .006 | .76 |
| LO1-AV2 | -219.37 | 32.64 | -6.72 | <.001 | .000 | .76 |
| LO1-AV3 | -240.63 | 31.72 | -7.59 | <.001 | .000 | .77 |
| LO1-HI3 | -327.52 | 42.43 | -7.72 | <.001 | .000 | .64 |
| LO1-HI2 | -448.81 | 43.98 | -10.21 | .000 | .000 | .64 |
| LO1-HI4 | -470.23 | 54.61 | -8.61 | .000 | .000 | .51 |
| LO1-HI1 | -534.35 | 46.83 | -11.41 | .000 | .000 | .62 |
| AV1-AV2 | -99.28 | 33.86 | -2.93 | .003 | .121 | .76 |
| AV1-AV3 | -120.54 | 32.98 | -3.66 | <.001 | .009 | .76 |
| AV1-HI3 | -207.44 | 43.38 | -4.78 | <.001 | .000 | .64 |
| AV1-HI2 | -328.72 | 44.89 | -7.32 | <.001 | .000 | .67 |
| AV1-HI4 | -350.14 | 55.35 | -6.33 | <.001 | .000 | .55 |
| AV1-HI1 | -414.26 | 47.69 | -8.69 | .000 | .000 | .66 |
| AV2-AV3 | -21.26 | 33.76 | -.63 | .529 | 1.000 | .75 |
| AV2-HI3 | -108.15 | 43.97 | -2.46 | .014 | .501 | .67 |
| AV2-HI2 | -229.44 | 45.47 | -5.05 | <.001 | .000 | .68 |
| AV2-HI4 | -250.86 | 55.82 | -4.49 | <.001 | .000 | .56 |
| AV2-HI1 | -314.98 | 48.23 | -6.53 | <.001 | .000 | .67 |
| AV3-HI3 | -86.90 | 43.30 | -2.01 | .045 | 1.000 | .63 |
| AV3-HI2 | -208.18 | 44.81 | -4.65 | <.001 | .000 | .64 |
| AV3-HI4 | -229.60 | 55.29 | -4.15 | <.001 | .001 | .51 |
| AV3-HI1 | -293.72 | 47.62 | -6.17 | <.001 | .000 | .62 |
| HI3-HI2 | 121.29 | 52.94 | 2.29 | .022 | .790 | .65 |
| HI3-HI4 | -142.70 | 62.06 | -2.30 | .021 | .773 | .85 |
| HI3-HI1 | 206.83 | 55.33 | 3.74 | <.001 | .007 | .56 |
| HI2-HI4 | -21.43 | 63.12 | -.34 | .734 | 1.000 | .72 |
| HI2-HI1 | 85.54 | 56.52 | 1.51 | .130 | 1.000 | .66 |
| HI4-HI1 | 64.12 | 65.14 | .98 | .325 | 1.000 | .62 |

*Note. U – U* Mann-Whitney statistic, *SE* – standard error, *z* – standardized U Mann-Whitney statistic, *p* – significance level, *p-*corrected – significance level with automatic Bonferroni correction, *η^2^* – effect size. LO – Low Impairment cluster, AV – Average Impairment cluster, HI – High Impairment cluster. Numbers reflect the number of a specific cluster, as described in the manuscript main body.

**Supplementary Material S5**

**Full list of measured variables in the whole project**

The order was fixed. The study was conducted in Polish.

- Demographic survey (own construction)
- Personality Inventory for ICD-11, Oltmanns & Widiger, 2018, Polish version: Cieciuch et al., 2021
- Level of Personality Functioning Scale, Weekers et al., 2019, Polish version: Łakuta et al., 2021
- Patient Health Questionnaire-9, Kroenke et al., 2001; Polish version: Kokoszka et al., 2016
- Hostile attribution bias measure – Ambiguous Visual Scenes Measure, Wilkowski et al., 2007; Polish version: Zajenkowska & Rajchert, 2020
- Ambiguous Intentions and Hostility Questionnaire, Combs et al., 2007, Polish version: Zajenkowska et al., 2018
- Green Paranoid Thoughts Scale - Revised (R-GPTS), Freeman et al., 2021, Polish version: Kowalski & Gawęda, n/d
- Experiences from childhood survey (own construction)
- Readiness to participate in clinical studies survey (not connected to actual invitation to do so; own construction)
